# Methylated Septin9 (m*SEPT9*): A Promising Blood-Based Biomarker for the Detection and Screening of Early-Onset Colorectal Cancer

**DOI:** 10.1158/2767-9764.CRC-21-0142

**Published:** 2022-02-11

**Authors:** Holli A. Loomans-Kropp, Yurong Song, Manish Gala, Aparna R. Parikh, Emily E. Van Seventer, Rocio Alvarez, Megan P. Hitchins, Robert H. Shoemaker, Asad Umar

**Affiliations:** 1 Cancer Prevention Fellowship Program, Division of Cancer Prevention, NCI, NIH, Rockville, Maryland.; 2 Gastrointestinal and Other Cancers Research Group, Division of Cancer Prevention, NCI, NIH, Rockville, Maryland.; 3 Cancer ImmunoPrevention Laboratory, Frederick National Laboratory for Cancer Research, Frederick, Maryland.; 4 Gastrointestinal Unit, Massachusetts General Hospital, Harvard Medical School, Boston, Massachusetts.; 5 Division of Hematology and Oncology, Massachusetts General Hospital, Harvard Medical School, Boston, Massachusetts.; 6 Biomedical Sciences, Cedars-Sinai Medical Center, Los Angeles, California.; 7 Chemopreventive Agent Development Research Group, Division of Cancer Prevention, NCI, NIH, Rockville, Maryland.

## Abstract

Early-onset colorectal cancer (EOCRC), defined as a diagnosis under age 50, is an emerging public health burden. As many of these individuals fall outside of screening guidelines, the development of a minimally invasive, accurate screening modality for this population is warranted. We evaluated the FDA-approved blood-based biomarker methylated Septin9 (m*SEPT9*) test as screening tool for EOCRC. EOCRC plasma, healthy plasma, and serum-free conditioned media from cancer cell lines were collected. Cell-free DNA (cfDNA) was isolated and bisulfite converted for use in the assay. m*SEPT9* and *ACTB* measured using Epi proColon V2.0. EOCRC plasma was collected at Massachusetts General Hospital (2005–2019) and controls were collected at the NIH and by ZenBio Inc. (prior to 2019). Twenty-seven EOCRC cases, 48 healthy controls <50 years old, and 39 healthy controls ≥50 years old were included in this study. m*SEPT9* was detected more frequently in EOCRC cases (88.9%) compared with healthy controls age <50 (4.2%) and ≥50 (15.4%), respectively (*P* < 0.001). The sensitivity, specificity, positive predictive value, and negative predictive values of the m*SEPT9* assay to detect EOCRC was 90.8% (95% CI, 84.7%–96.9%), 88.9% (95% CI, 77.0%–100.0%), 96.3% (95% CI, 92.3%–100.0%), and 75.0% (95% CI, 60.0%–90.0%), respectively, compared with all healthy controls. m*SEPT9* cfDNA level was an independent predictor of survival (*P* = 0.02). m*SEPT9* is a sensitive and specific biomarker for EOCRC detection. These results suggest that m*SEPT9* may be useful in the detection of EOCRC, providing a minimally invasive method for screening in this growing population of patients with colorectal cancer.

**Significance:**

m*SEPT9* may be a novel biomarker for the detection of early-onset colorectal cancer, as it demonstrated high sensitivity and specificity in our study.

## Introduction

Incidence of early-onset colorectal cancer (EOCRC), defined as a colorectal cancer diagnosis under the age of 50 years, has dramatically increased over the last several decades in the United States and globally (1). The risk factors contributing to the rising trends of EOCRC remain undefined, although several factors, such as increased obesity, dietary changes, and sedentary lifestyle, have been proposed (2–4). Despite the increasing incidence, unless there is a known genetic predisposition, most individuals with EOCRC are not screened until they are symptomatic. Although prominent gastroenterological societies have begun recommending endoscopic screening at age 45, the influx of new screen-eligible individuals will be difficult to manage given systemic constraints, workforce shortages, and the high cost of implementation (5, 6). Therefore, creating solutions for this unforeseen issue needs to be prioritized.

Despite an increase in the incidence of colorectal cancer in individuals under the age of 50 years, as a proportion of all colorectal cancer cases, EOCRC is still small and widespread screening of this age group may not be the most feasible or cost-effective strategy. However, a tiered screening strategy with the use of a less-invasive approach like fecal immunochemical test (FIT), Cologuard, or a blood-based test has been proposed (6). The integration of a sensitive and specific blood-based assay may fill the EOCRC screening and detection gap. Plasma-based circulating biomarkers, such as cell-free DNA (cfDNA), are often used for the detection of somatic alterations in cancer and sensitive modalities for its detection have been recently developed (7–10). The use of cfDNA for cancer detection is promising, as tumor-derived cfDNA is abundant compared with normal circulating cfDNA and remains relatively stable during long-term storage (11–13). This has been established in lung, prostate, breast, and colorectal cancers (14–17). Furthermore, the addition of blood-based biomarkers, such as methylated *SEPT9* (m*SEPT9*), to FIT has demonstrated improved overall screening sensitivity (18–20).

m*SEPT9* has displayed efficacy as a plasma-based circulating biomarker for the detection of colorectal cancer, as *SEPT9* production is regulated by epigenetic events which have proven critical in the initiation and progression of cancer (21, 22). Moreover, m*SEPT9* can be easily and reliably detected in plasma collected from tumor-bearing individuals (23, 24). Furthermore, numerous clinical studies have demonstrated high sensitivity and specificity of m*SEPT9* for the detection of colorectal cancer (23, 25, 26). A recent meta-analysis of published case–control studies evaluating the performance of m*SEPT9* showed a pooled sensitivity of 74% (95% CI, 61%–84%) and specificity of 84% (95% CI, 81%–87%), comparing colorectal cancer to healthy individuals (27). These and other studies provided compelling evidence to grant FDA approval for Epi proColon, a commercially available m*SEPT9* detection kit (28). Epi proColon is the only FDA-approved blood-based screening tool for colorectal cancer; however, its approval is limited to individuals age 50 and older who have refused colonoscopy or fecal-based screening methods (29, 30). Therefore, in this study, we seek to extend the population utility of Epi proColon.

Because of the increasing trend in EOCRC and the significant burden on the health care system for colorectal cancer screening, a rapid, noninvasive modality to triage potential EOCRC cases is needed. However, no studies have evaluated the efficacy of m*SEPT9* as a colorectal cancer screening modality in a younger population. In this study, we evaluated the efficacy of the commercially available m*SEPT9* assay, Epi proColon V2.0, for the detection of colorectal cancer in a retrospective case–control study of archived EOCRC plasma samples, compared with control plasma collected from healthy individuals <50 years and healthy controls ≥50 years. We hypothesized that m*SEPT9* would be a sensitive and specific biomarker for EOCRC detection in this cohort, comparable with that reported for individuals ≥50 years old for which Epi proColon is FDA approved.

## Materials and Methods

### Plasma Collection, Preparation, and Patient Information

Plasma from cases with an EOCRC diagnosis under age 50 and healthy (disease-free at time of blood collection) controls younger than age 50 at time of collection were used in this study. All EOCRC plasma samples were treatment-naïve and collected prior to surgery. The study protocol and use of biospecimens were reviewed and determined exempt by the NIH Institutional Review Board. Healthy donor blood was acquired from the NIH Clinical Center (CC) and a commercial vendor (ZenBio, Inc.). Blood acquired through the NIH CC was collected into EDTA Vacutainer tubes and transported Frederick National Laboratory for Cancer Research (FNLCR). Upon arrival, blood samples were spun for 10 minutes at 500 × *g*. The top layer (plasma) was transferred and pooled into a 15 mL conical tube and spun at 2,000 × *g* for 10 minutes. Plasma was stored in 0.5-mL aliquots at −80°C until DNA extraction. Similarly, plasma procured from the commerical vendor was collected in EDTA Vacutainer tubes and processed and stored according to the manufacturer's specifications.

EOCRC plasma samples were collected at Massachusetts General Hospital between May 2005 and February 2019. Briefly, venous blood was collected by standard phlebotomy into EDTA Vacutainer tubes and sent for processing. Upon arrival, samples were centrifuged at 1,600 × *g* for 10 minutes and plasma supernatant transferred to a 15 mL centrifuge tube for an additional centrifugation step for 10 minutes at 3,000 × *g*. The plasma was transferred to a fresh 15 mL tube, gently mixed, and stored in 1-mL aliquots. Aliquots were stored at −80°C at Massachusetts General Hospital (Boston, MA) until shipment on dry ice to FNLCR, where they were stored at −80°C upon arrival. All EOCRC cases included in the study had biopsy-confirmed colorectal cancer. Controls were healthy (cancer free) at the time of collection and acquired from the NIH (2018–2019) and the commercial vendor ZenBio Inc. (prior to 2019). Demographic, diagnostic, and prognostic information of EOCRC cases and demographic information of controls were collected. All samples were deidentified.

EOCRC sample collection was approved by the Massachusetts General Hospital Institutional Review Board (IRB 14–046) and the study was deemed exempt from NIH Institutional Review Board approval (IRB000294). Written informed consent from all participants was obtained at their respective collection sites. The study was conducted in accordance with the U.S. Common Rule.

### Cell Culture

Colorectal cancer cell lines HCT-116 (obtained 2018), HT-29 (obtained 2018), and LoVo (obtained 2018), lung cancer cell line HOP-92 (obtained 2017), breast cancer cell line MCF7 (obtained 2021), and melanoma cell line RPMI-7951 (obtained 2021) were obtained through the NCI Cell Line Repository [Division of Cancer Treatment and Diagnosis (DCTD) Tumor Repository, NCI at Frederick, Frederick, MD]. The prostate cancer cell line PC-3 was provided by Dr. Esta Sterneck (NCI-Frederick, obtained 2019). HCT-116, HT-29, LoVo, HOP-92, MCF7, RPMI-7951, and PC-3 cells were cultured in RPMI1640 medium supplemented with 10% FBS, 1% penicillin/streptomycin, and 2 mmol/L l-glutamine. All cells were incubated at 5% CO_2_ at 37°C. Cell lines were tested for *Mycoplasma* contamination by PPLO culture under aerobic and anaerobic conditions and orcein staining of Fogh or by PCR-based assay. Cell lines obtained from the NCI DCTD Tumor Repository (HCT-116, HT-29, LoVo, HOP-92, MCF7, RPMI-7951) were authenticated using Applied Biosystems AmpFISTR Identifiler with PCR amplification prior to cell line receipt. PC-3 cells were authenticated using CellCheck (IDEXX BioAnalytics), a comprehensive cell line authentication service that utilizes STR-based DNA profiling and multiplex PCR to detect both contamination and misidentification of cell lines.

### Serum-Free Conditioned Media Collection

Cell lines were thawed according to repository guidelines. Passage number between thawing and serum-free conditioned media (SFCM) collection was kept to a minimum. Cells were grown to 90% confluence in 75 mm^2^ flasks, washed with 3 mL 1x DPBS, and serum- and antibiotic-free media were added to the cells and incubated at 37°C overnight. SFCM was collected, centrifuged briefly to rid of cellular debris, and stored in 1 mL aliquots at −80°C until use.

### Epi ProColon V2.0 Assay Kit

The Epi proColon V2.0 plasma circulating cfDNA test kit protocol was performed according to the manufacturer's protocol, however, adapted to a smaller sample volume (1 mL), as demonstrated in Hitchins and colleagues 2019 (31). Briefly, 1 mL plasma and assay controls were thawed at room temperature for 30 minutes. Samples were transferred to a 15 mL conical tube and 1 mL Epi proColon Lysis Binding Buffer added, briefly vortexed, and incubated at room temperature for 10 minutes. Following incubation, 25.7 μL magnetic beads and 714 μL molecular grade absolute ethanol was added to each sample, then mixed by inversion and rotated for 45 minutes to complete DNA binding. Upon completion, samples were incubated at 56°C for 10 minutes, washed with 500 μL Epi proColon Wash Buffer A, and bound DNA eluted into 50 μL Epi proColon Elution Buffer. Next, bisulfite conversion was performed by adding 75 μL Epi proColon Bisulfite and 12.5 μL Epi proColon Protection Buffer to the extracted DNA. Samples were briefly vortexed, spun down, and incubated at 80°C for 45 minutes. Immediately after incubation, samples were briefly spun down and 500 μL Epi proColon Wash Buffer A and 10 μL Epi proColon Magnetic Beads were added to complete DNA binding. Samples were briefly vortexed, centrifuged, and incubated at 23°C while shaking at 1,000 rpm. The magnetic bead solutions were then centrifuged and placed in a magnetic rack to remove the remaining buffer. The bound beads were washed three times, first with 500 μL Epi proColon Wash A Buffer, and subsequently with 400 μL and 200 μL Epi proColon Wash B. After removing all wash buffer, the beads were dried at 23°C for 10 minutes and bisulfite-converted DNA (bisDNA) eluted into 17 μL of Epi proColon Elution Buffer. Internal positive and negative controls were included in each batch (Epi proColon Sensitive PCR Kit, Epigenomics, Inc.).

SFCM volumes of 1 mL, 500 μL, 250 μL, and 125 μL were used for volume titration of the Epi proColon V2.0 kit. Samples were diluted with 1x DPBS to a volume of 1 mL then processed in the same manner as the plasma samples.

### Quantitative PCR

Immediately following the isolation of bisDNA, the samples were randomized in batches and analyzed by qPCR using the Epi proColon Sensitive PCR Kit. A volume of 15 μL of PCR Master Mix was added to 15 μL of bisDNA and the plate was briefly centrifuged. All samples were run using an Applied Biosystems QuantStudio 5. Thermal cycle program conditions were as follows: (i) denaturation for 20 minutes at 94°C (40% ramp rate); (ii) annealing and extension for 5 seconds at 62°C (80% ramp rate), 35 seconds at 55.5°C (80% ramp rate), and 30 seconds at 93°C (40% ramp rate) for 45 cycles; and (iii) extension for 5 seconds at 40°C (80% ramp rate). A valid assay run had positive control m*SEPT9* and *ACTB* thresholds less than cycle threshold (*C*_t_) ≤ 41.4 and *C*_t_ ≤ 29.8, respectively, and negative control m*SEPT9* and *ACTB* thresholds undetermined and *C*_t_ ≤ 37.2, respectively. Patient plasma samples were considered positive if *ACTBC*_t_ ≤ 32.1 and m*SEPT9C*_t_ < 45, negative if *ACTBC*_t_ ≤ 32.1 and m*SEPT9* undetermined, and invalid *ACTBC*_t_ > 32.1.

### Statistical Analysis

As the protocol was adapted to 1 mL plasma (1/3 of the original protocol volume), a single real-time PCR reaction was performed in a single well for each sample. m*SEPT9* positivity was determined using the 1/1 testing algorithm, whereby if the result for m*SEPT9* and internal *ACTB* reached the specified threshold, then the sample was considered positive. If the assay and sample controls passed quality control, sample m*SEPT9* levels were evaluated. If m*SEPT9* was detected below a *C*_t_ of 45, the sample was determined positive. Each case and control was analyzed with a dichotomous (positive, negative) outcome and relative methylation was determined using the ∆∆*C*_t_ method for DNA methylation, as described elsewhere (32, 33). Receiver operating curves (ROC) were generated using qPCR *C*_t_ values. Statistical differences in relative methylation were determined by one-way ANOVA or Mann–Whitney *U* test. A *P* value less than 0.05 was considered statistically significant. Analyses were performed in GraphPad Prism 8 for Windows (GraphPad Software, Inc.) and sensitivity, specificity, positive predictive value (PPV), and negative predictive value (NPV) were calculated in SAS 9.4 (SAS Institute).

### Data Availability

The datasets generated and/or analyzed during this study are not publicly available due to the sensitivity of the data but are available from the corresponding author upon reasonable request.

### Ethics Approval and Consent to Participate

The collection of samples included in this study was approved by the Massachusetts General Hospital Institutional Review Board (IRB 14–046) and the study was deemed exempt from NIH Institutional Review Board approval (IRB000294).

## Results

The study cohort included 34 EOCRC cases, 50 healthy controls <50 years old, and 40 healthy controls ≥50 years old. Of these, 10 samples were excluded due to failed tests. The final cohort for which complete data were obtained included 27 EOCRC cases, 48 healthy controls <50 years old, and 39 healthy controls ≥50 years old (114 total). EOCRC cases had a median age of 44 years (range 25.9–49), were 81% white and 59% male (Table 1). Healthy controls <50 years old had a median age of 44 (range 29–49), were 48% black and 65% male, while healthy controls ≥50 years old had a median age of 56 (range 50–77), were 54% white and 64% male. Majority of the EOCRC cases were rectal cancers (66.7%), late stage (62.9% stage III/IV), and had a family history of cancer (77.8%; Table 2).

**TABLE 1:** Demographics of healthy controls <50 years old, healthy controls ≥50 years old, and EOCRC cases.

Variable	Healthy controls <50 years (*N* = 48)	Healthy controls ≥50 years (*N* = 39)	EOCRC (*N* = 27)
Age (median, range)	44 (29–49)	56 (50–77)	44 (25.9–49)
Race (*n*, %)			
White	9 (18.8)	21 (53.8)	22 (81.5)
Black	23 (47.9)	4 (10.3)	2 (7.4)
Hispanic	15 (31.3)	7 (17.9)	0 (0.0)
Asian	1 (2.1)	5 (12.8)	1 (3.7)
Unknown	0 (0.0)	1 (2.6)	2 (7.4)
Sex (*n*, %)			
Female	17 (35.4)	14 (35.9)	11 (40.7)
Male	31 (64.6)	25 (64.1)	16 (59.3)
mSEPT9 assay (*n*, %)			
Positive	2 (4.2)	6 (15.4)	24 (88.9)
Negative	46 (95.8)	33 (84.6)	3 (11.1)

**TABLE 2:** Clinical characteristics of EOCRC cases.

Variable	EOCRC (*N* = 27)
Cancer site (*n*, %)	
Colon	3 (11.1)
Rectum	18 (66.7)
Unspecified colorectal site	6 (22.2)
Stage (*n*, %)	
I	4 (14.8)
II	1 (3.7)
III	5 (18.5)
IV	12 (44.4)
Unknown	5 (18.5)
Tumor grade (*n*, %)	
Low, low/intermediate	11 (40.7)
Intermediate	6 (22.2)
High	7 (25.9)
Unknown	3 (11.1)
Survival status (*n*, %)	
Alive	21 (77.8)
Deceased	6 (22.2)
Family history of cancer (*n*, %)	
Yes	21 (77.8)
No	5 (18.5)
Unknown	1 (3.7)
Family history of CRC (*n*, %)	
Yes	9 (33.3)
No	17 (63.0)
Unknown	1 (3.7)
History of IBD or chronic inflammation (*n*, %)	
Yes	5 (18.5)
No	22 (81.5)

Abbreviations: CRC, colorectal cancer; IBD, inflammatory bowel disease.

**FIGURE 1:**
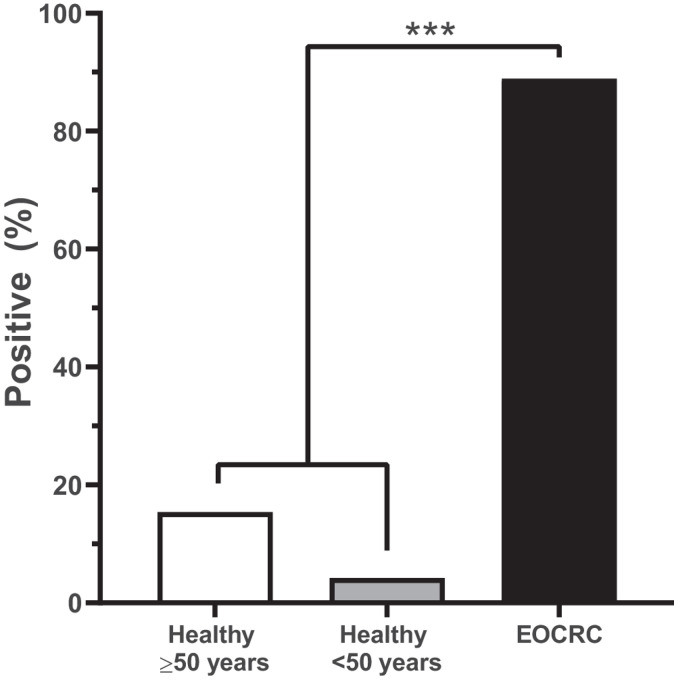
EOCRC cases showed significantly higher m*SEPT9* positivity than healthy controls. Significantly more EOCRC cases (colorectal cancer ≤50 years) were m*SEPT9* positive, compared with plasma from healthy controls <50 years old and ≥50 years old (*P* < 0.001).

Abiding by the thresholds established in the Epi proColon V2.0 kit, significantly more EOCRC samples were positive for m*SEPT9* compared with healthy controls <50 years and healthy controls ≥50 years (*P* < 0.001; Fig. 1). *ACTB* values were not statistically different between EOCRC cases and healthy controls (*P* = 0.53). Specifically, 4.2% (2/48) of healthy controls <50 years old, 15.4% (6/39) of healthy controls ≥50 years old, and 88.9% (24/27) of EOCRC cases were positive for m*SEPT9*. Interestingly, no healthy samples under the age of 40 were m*SEPT9* positive, and the highest percentage of m*SEPT9*-positive healthy controls was the in 50- to 55-year age group (21%; Table 3).

**TABLE 3:** m*SEPT9* status by demographics of EOCRC cases and healthy controls.

Variable	Healthy controls <50 years (*N* = 48)	Healthy controls ≥50 years (*N* = 39)	EOCRC (*N* = 27)
*Age* (*n*, %)			
26–29	0/1 (0.0)	–	1/1 (100.0)
30–34	0/1 (0.0)	–	1/1 (100.0)
35–39	0/4 (0.0)	–	3/3 (100.0)
40–44	1/20 (5.0)	–	10/11 (90.9)
45–49	1/23 (4.3)	–	9/11 (81.8)
50–54	–	3/17 (17.6)	–
55–59	–	1/7 (14.3)	–
60–64	–	1/8 (12.5)	–
65–69	–	1/5 (20.0)	–
70+	–	0/2 (0.0)	–
Race (*n*, %)			
White	1/9 (11.1)	4/21 (19.0)	19/22 (86.3)
Black	1/23 (4.3)	0/5 (0.0)	2/2 (100.0)
Hispanic	0/15 (0.0)	2/7 (28.6)	0/0 (0.0)
Asian	0/1 (0.0)	0/5 (0.0)	1/1 (100.0)
Unknown	0/0 (0.0)	0/1 (0/0)	2/2 (100.0)
Sex (*n*, %)			
Female	0/17 (0.0)	2/14 (14.3)	11/11 (100.0)
Male	2/21 (9.5)	4/25 (16.0)	13/16 (81.3)
Cancer site (*n*, %)			
Colon	–	–	3/3 (100.0)
Rectum	–	–	15/18 (83.3)
Unspecified colorectal site	–	–	6/6 (100.0)
Stage (*n*, %)			
I	–	–	4/4 (100.0)
II	–	–	1/1 (100.0)
III	–	–	5/5 (100.0)
IV	–	–	12/12 (100.0)
Unknown	–	–	2/5 (40.0)
Tumor grade (*n*, %)			
Low, low/intermediate	–	–	10/11 (90.9)
Intermediate	–	–	5/6 (83.3)
High	–	–	6/7 (85.7)
Unknown	–	–	3/3 (100.0)
Survival status, overall (*n*, %)			
Alive	–	–	18/21 (85.7)
Deceased	–	–	6/6 (100.0)
Family history of cancer (*n*, %)			
Yes	–	–	19/21 (90.5)
No	–	–	4/5 (80.0)
Unknown	–	–	1/1 (100.0)
Family history of CRC (*n*, %)			
Yes	–	–	8/9 (88.9)
No	–	–	15/17 (88.2)
Unknown	–	–	1/1 (100.0)
History of IBD or chronic inflammation (*n*, %)			
Yes	–	–	5/5 (100.0)
No	–	–	19/22 (86.4)

Abbreviations: CRC, colorectal cancer; IBD, inflammatory bowel disease.

**FIGURE 2:**
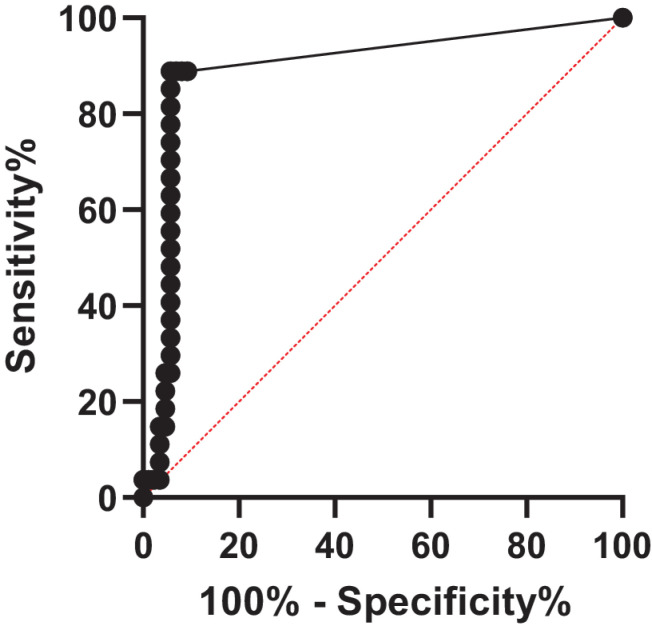
ROC of m*SEPT9* comparison between EOCRC cases and healthy controls. The ROC was generated by comparing the m*SEPT9 C*_t_ values of EOCRC cases and all healthy controls. The AUC (0.89) was statistically significant (SE, 0.04; 95% CI, 0.81–0.97; *P* < 0.001).

m*SEPT9* was detected at similar frequency in EOCRC stages I–IV. Additional control and EOCRC demographics are reported in Table 3, as well as m*SEPT9* positivity by EOCRC clinicopathologic characteristics. The overall sensitivity (for EOCRC of all stages I–IV), specificity, PPV, and NPV of the m*SEPT9* assay were calculated to be 90.8% (95% CI, 84.7%–96.9%), 88.9% (95% CI, 77.0%–100.0%), 96.3% (95% CI, 92.3%–100.0%), and 75.0% (95% CI, 60.0%–90.0%), respectively. ROC curves were generated to evaluate the performance of the assay in distinguishing colorectal cancer cases from non–colorectal cancer controls (healthy controls ≤50 and >50 years old, combined). Defining colorectal cancer cases as positive and non–colorectal cancer healthy controls as negative produced an area under the curve (AUC) of 0.89 (95% CI, 0.81–0.97; *P* < 0.001), suggesting that the m*SEPT9* assay can sensitively and specifically distinguish colorectal cancer from non–colorectal cancer (Fig. 2).

**FIGURE 3:**
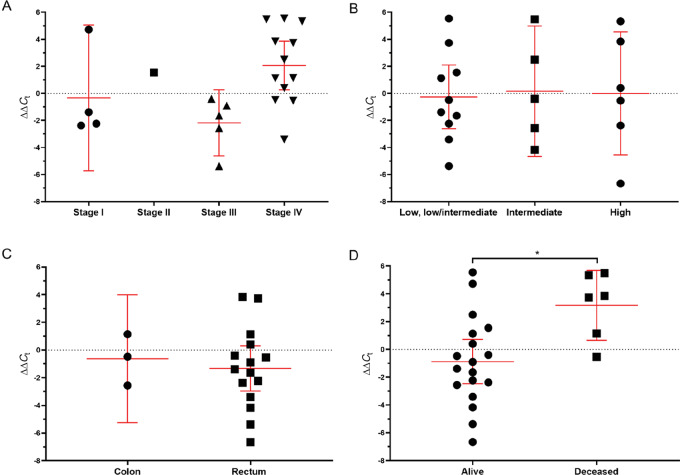
Presence of m*SEPT9* was significantly associated with EOCRC survival status. Comparison of presence of m*SEPT9*, normalized by batch and *ACTB*, by stage (**A**; *P* = 0.06, *P*_trend_ = 0.13), tumor grade (**B**; *P* = 0.98, *P*_trend_ = 0.88), tumor site (**C**; *P* = 0.65), and survival status (**D**; *P* = 0.02).

We next decided to quantitatively evaluate the positive EOCRC cases, normalizing sample *C*_t_ values to the within batch controls (∆∆*C*_t_). No significant differences in ∆∆*C*_t_ were noted between stages (*P* = 0.06, *P*_trend_ = 0.13), tumor grade (*P* = 0.98, *P*_trend_ = 0.88), or tumor site (*P* = 0.65; Fig. 3A–C). However, we did observe a significant difference in patient outcome. EOCRC cases with a follow-up status of deceased had significantly greater levels of plasma m*SEPT9* (∆∆*C*_t_) compared with cases with a follow-up status of alive (*P* = 0.02), suggesting m*SEPT9* plasma levels are prognostic (Fig. 3D). Overall, among positive EOCRC cases, level of plasma m*SEPT9* was an independent predictor of overall survival.

**FIGURE 4:**
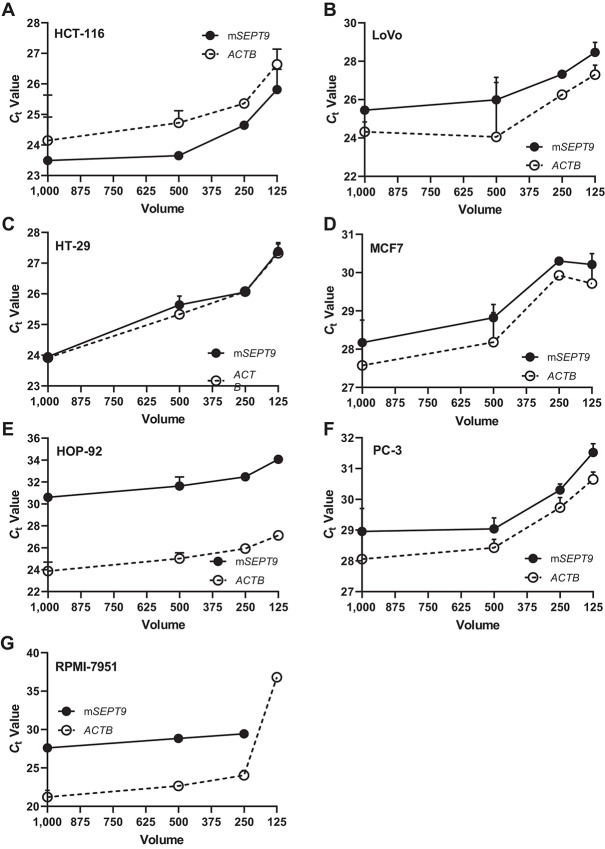
m*SEPT9* was detectable in small volumes of SFCM in colorectal cancer and non–colorectal cancer cell lines. Volume titration of SFCM from colorectal cancer and non–colorectal cancer cell lines. m*SEPT9* and *ACTB* detection was evaluated in 1,000 μL, 500 μL, 250 μL, and 125 μL of SFCM in HCT-116 (**A**), LoVo (**B**), HT-29 (**C**), MCF7 (**D**), HOP-92 (**E**), PC-3 (**F**), and RPMI-7951 (**G**).

We and others have established the ability of the Epi proColon V2.0 kit to detect m*SEPT9* in small volumes of plasma collected from individuals with colorectal cancer; however, the production of m*SEPT9* as a biomarker in additional cancer types remains unexplored. We evaluated conditioned media collected from cancer cell lines. We collected SFCM from cell culture (HCT-116, LoVo, HT-29, HOP-92, PC-3, MCF7, RPMI-7951) for bisDNA conversion. m*SEPT9* and *ACTB* was in the detectable range in the SFCM of most cell lines, down to a volume of 125 μL (Fig. 4A–G). m*SEPT9* was not detectable in 125 μL of melanoma RPMI-7951 SFCM, although *ACTB* was present (Fig. 4G). *C*_t_ values for m*SEPT9* and *ACTB* were within the same range for colorectal cancer, prostate, lung, breast, and melanoma cancer cell lines. For all cell lines, *C*_t_ values were similar between 500 μL and 1 mL of SFCM.

## Discussion

In this study, we found that plasma m*SEPT9* was specific and sensitive for the detection of EOCRC. EOCRC cases were found more frequently positive for m*SEPT9*, compared with healthy controls <50 years and healthy controls ≥50 years. Furthermore, we were able to detect consistently and accurately m*SEPT9* in samples using a small plasma volume (1 mL) and measurement in a single real-time PCR reaction (31). To our knowledge, this is the first evaluation of the utility of m*SEPT9* as a screening modality in the early-onset population. Previous investigations of m*SEPT9* among individuals of screening age (≥50 years old) and in individuals with Lynch syndrome, have demonstrated the potential of m*SEPT9* as a sensitive and specific blood-based biomarker for colorectal cancer (24, 26, 31, 32, 34). Our study adds to this growing evidence base supporting the expansion of m*SEPT9* as a biomarker for colorectal cancer detection in the population under 50 years of age.

Using methylation for early detection of cancer can be challenging, as epigenetic markers accumulate along CpG islands with increasing age and over time (35–37). Some methylation changes associated with aging are predictable, such as methylation of ELOVL2, which is considered one of the most robust biomarkers associated with age, and methylation profiles differ between aging and cancer (38, 39). Methylation of *SEPT9* has not been described in aging profiles, suggesting its specificity to cancer. In addition to colorectal cancer, m*SEPT9* has been associated with overall survival in head and neck squamous cell carcinoma, cholangiocarcinoma, lymph node status in bladder cancer, nonbasal breast cancer, and lung cancer (40–44). Despite the prognostic implications, m*SEPT9* has been moved forward as a diagnostic biomarker for colorectal cancer (45).

EOCRC is a rising public health problem in the United States and globally (46, 47). However, the majority of these younger individuals fall outside of the current screening guidelines. Furthermore, initiating screening by colonoscopy or sigmoidoscopy at earlier ages would place additional burden on an already overwhelmed system (48). Alternative approaches that are more accessible and cost-effective, including blood-based screening, provides an opportunity to fill this notable screening gap. On a population scale, blood-based approaches, such as Epi proColon, could be used to triage individuals under the age of 50, prior to receiving a colonoscopy.

A limitation to the current Epi proColon assay is the evaluation of a single blood-based biomarker, although m*SEPT9* demonstrated high sensitivity and specificity. Using a multiplexed platform could improve the diagnostic ability of m*SEPT9*. For example, an evaluation of *KISS1R, SEPT9,* and *CSAD* methylation in bladder cancer improved the AUC for predicting lymph node status (AUC = 0.68–0.72), compared with *KISS1R* (AUC = 0.67) *SEPT9* (AUC = 0.58), or *CSAD* (AUC = 0.70) alone (42). Moreover, utilizing a multimarker blood-based approach may afford an opportunity to simultaneously screen for multiple cancers; however, this would require the identification of organ-specific gene or methylation signatures. Multigene or methylation panels may soon allow for this type of approach (49). An additional limitation of note is that EOCRC cases and controls were not collected simultaneously. Cases and controls were, however, processed using almost identical protocols and stored without freeze/thaw at −80°C until used in the m*SEPT9* assay. Therefore, we are confident that all caution was taken to handle the biospecimens the same despite different collection locations. Finally, it is important to note that this study was limited in its sample size. Although EOCRC incidence is rising, availability of samples remains limited. Even though the EOCRC sample size was small, we were able to observe strong associations between m*SEPT9* and different outcomes, which would only be strengthened with a larger study population.

Although this study provided new evidence in support of the utility of m*SEPT9* for the detection of EOCRC, our study had additional limitations to note. First, we conducted a case–control study using archival plasma samples with known cancer outcomes. Although incidence is increasing, EOCRC is infrequent and prospective collection is difficult. The use of archival biospecimens allowed for the current analysis extending m*SEPT9* detection into EOCRC. We are confident that the results obtained in our archival cohort reflect what would be measured in a fresh collection, as several studies have demonstrated that circulating tumor DNA remains stable with long-term storage (50) and is concordant with tumor tissue profiles (13). The results of this study provide support for the institution of a prospective EOCRC cohort (of greater than average risk) to thoroughly evaluate m*SEPT9* as a screening tool using freshly collected plasma. Next, the number of available EOCRC cases was limited. The participant pool was restricted to colorectal cancer–confirmed individuals under the age of 50 who were treatment naïve, as the effect of chemotherapy and radiation on m*SEPT9* is unknown. This limited the number of biospecimens available in the Massachusetts General Hospital biorepository. We additionally did not choose to extend our search for biospecimens to additional biorepositories or archival collections, in an attempt to limit the variability in sample collection and storage. Designing the case–control study in this manner limited the number of included samples. Despite the small sample size, we were able to observe significant differences in m*SEPT9* detection between EOCRC cases and healthy controls, as well as significant associations with clinical characteristics. This is the first evaluation of m*SEPT9* for the detection of colorectal cancer in this population, lending novelty to the analysis despite limited cases. We anticipate that expanding the study, or a subsequent study, to include more cases will strengthen our findings.

The strengths of the study are not without mention. We showed that m*SEPT9* could be detected at high sensitivity and specificity in 1 mL of plasma from both EOCRC cases and healthy controls, and in cell line SFCM at increasingly small volumes, highlighting the feasibility of this assay in a clinical setting where limited biospecimens are available. It is possible that *mSEPT9* will perform even better in optimal clinical screening settings. Furthermore, we were able to quantitatively measure m*SEPT9* in non–colorectal cancer cell lines, suggesting that m*SEPT9* may have applicability as a biomarker in other cancer types, recent data suggests this to be the case for esophageal, gastric, and liver cancer (51, 52). Future studies could focus on a pan-cancer evaluation of m*SEPT9* combined with organ-specific markers to distinguish the biomarker origin.

In conclusion, we demonstrated that m*SEPT9* is a sensitive and specific biomarker for the detection of colorectal cancer among individuals age under 50 years. Because of the increasing public health concern of EOCRC, the development of noninvasive screening modalities is warranted. Current research suggests that the detection of m*SEPT9* in plasma may help fill this gap. Additional studies are essential to develop and improve EOCRC screening modalities.
